# Incident of Office Practice

**Published:** 1902-02

**Authors:** John S. Engs

**Affiliations:** Oakland, Cal.


### Domestic Correspondence.

### INCIDENT OF OFFICE PRACTICE.

OAKLAND, CAL., December 18, 1901.

TO THE EDITOR:

SIR,—I enclose a little incident of office practice:

After the completion of a very protracted gold-filling operation, my patient, a skilled accountant, said that he had been keeping tally on the number of blows I used with my Snow and Lewis automatic, after applying each pellet of H. & R. No. ½ A gold. The number ranged from thirty-one to fifty with an average of thirty seven.

This may be of interest to those who are considering the force necessary to properly consolidate a gold filling. For my part I was quite unconscious of the blows, and used only what I thought necessary to properly consolidate the filling.

Very truly yours,

JOHN S. ENGS.

